# Comparing the Effect of Oral Clonidine and Tranexamic Acid on Bleeding and Surgical Field Quality during Functional Endoscopic Sinus Surgery

**Published:** 2018-09

**Authors:** Jahangir Ghorbani, Shima Arastou, Ali Safavi Naeini, Nasim Raad, Mahboobeh Karimi Galougahi, Alireza Jahangirifard, Nader Akbari Dilmaghani

**Affiliations:** 1 *Chronic Respiratory Disease Research Center, National Research Institute of Tuberculosis and Lung Diseases, Shahid Beheshti University of Medical Sciences, Tehran, Iran.*; 2 *Department of Rhinology, Shahid Beheshti University of Medical Sciences, Tehran, Iran.*; 3 *Tracheal Research Center, National Research Institute of Tuberculosis and Lung Diseases, Shahid Beheshti University of Medical Sciences, Tehran, Iran.*; 4 *Lung Transplantation Research Center, National Research Institute of Tuberculosis and Lung Diseases, Shahid Beheshti University of Medical Sciences, Tehran, Iran.*; 5 *Hearing Disorders Research Center, Shahid Beheshti University of Medical Sciences, Tehran, Iran.*

**Keywords:** Bleeding, Clonidine, Polyposis, Rhinosinusitis, Sinus, Surgery, Tranexamic acid

## Abstract

**Introduction:**

Bleeding during functional endoscopic sinus surgery (FESS) is an important issue for both anesthesiologists and surgeons as it can affect the safety and efficiency of the procedure. We compared the efficacy of tranexamic acid (TXA) and clonidine in reducing blood loss and improving surgical field visualization during FESS.

**Materials and Methods:**

In a double-blind, randomized, clinical trial, 52 patients, American Society of Anesthesiologists (ASA) physical status 1–2, aged 13–75 years, suffering from rhinosinusitis with or without polyposis, and who were candidates for FESS, were enrolled. The first group received intravenous TXA 15 mg/kg diluted in 100 ml normal saline, administered during 10-min infusion after induction. In the second group, 0.2 mg oral clonidine was given 1 to 1.5 hours before surgery. Duration of surgery, hemoglobin level, heart rate, blood pressure, and quality of surgical field based on Boezzart's scale and surgeon satisfaction based on Likert's scale were recorded in both groups.

**Results:**

In total, 52 patients, 27 (51.9%) males and 25 (48.07%) females were studied. Twenty-two (42.3%) and 30 (57.7%) were in the TXA and clonidine groups, respectively. The mean pre- and post-surgical hemoglobin level showed no meaningful difference between the two groups. The same result was obtained for blood pressure and heart rate at different time points (P>0.05). Mean anesthesia time (P=0.859), mean surgical time (P=0.880), surgeon's satisfaction of the surgical field (P=0.757) and surgical field quality at different time points revealed no significant difference between the two groups.

**Conclusion:**

Premedication with oral clonidine and intravenous TXA has the same effect on bleeding during FESS, surgical field visualization, and surgeon satisfaction.

## Introduction

Bleeding during functional endoscopic sinus surgery (FESS) is an important issue for both anesthesiologists and surgeons (1), due to the high vascularity of the sinuses and nasal mucosa (2). Despite the low rate of major bleeding during FESS, maintaining maximal visibility of the surgical field for the surgeon is of great importance (1). In some cases, even small amounts of blood can affect the endoscopic view, resulting in possible complications, extended or even incomplete surgery, and therefore a lower success rate (1,2). To date, many methods have been proposed for improving the surgical field in sinus surgery; packing, bipolar cauterization, local vasoconstrictors, patient head elevation, use of medications (e.g. beta blockers), and controlled hypotensive anesthesia are among the most commonly used techniques (1,3). Furthermore, many drugs have been applied to induce hypotensive anesthesia. Clonidine, an alpha2-adrenergic agonist, is one of the medications used for controlling tachycardia, blood pressure (BP), bleeding, postoperative pain and vomiting in many surgical fields (4). Following oral administration, clonidine is rapidly absorbed, and its concentration rises to a maximum level in 1.5 to 2 hours; its half-life is around 8-12 hours (5). Oral clonidine enhances surgical field visualization by reducing intraoperative bleeding and is very effective in increasing surgeon satisfaction during FESS (6–8).Tranexamic acid (TXA) is a synthetic derivative of the lysine amino acid; it inhibits lysine-binding sites on plasminogen molecules resulting in fibrinolysis blockage (2,3). Intravenous TXA has been shown to effectively reduce bleeding as well as improve surgical field visualization during FESS(9-14). However, the systemic administration of TXA can lead to adverse events, including dizziness, blurred vision, nausea, vomiting, and headache. Interestingly, there is still no evidence regarding an increased risk of thrombosis in major surgeries (2).

As no study has yet compared oral clonidine and intravenous TXA, our objective was to compare their effect as premedication on parameters such as hemodynamic changes during FESS, duration of surgery, level of surgeon satisfaction, and surgical field quality (4).

## Materials and Methods

This double-blind, randomized clinical trial was conducted from October 2016 to June 2017. Fifty-two patients with an American Society of Anesthesiologists (ASA) Physical Status Classification of 1 or 2 (a normal healthy patient or a patient with mild systemic disease), aged 13 to 75 years, suffering from rhinosinusitis with or without polyposis visiting the ear, nose and throat (ENT) clinic of Masih Daneshvari Hospital, Tehran, Iran, who were candidates for endoscopic sinus surgery, were enrolled. The study protocol was approved by the Research Ethics Committee of Shahid Beheshti University of Medical Sciences and written informed consent was obtained from all patients prior to study entrance.

Patients with the following criteria were excluded from the study: history of previous sinonasal surgery, underlying disease with increased risk of thrombosis, chronic systemic disease that would not allow controlled hypotension, those using calcium channel blockers, anticoagulants, digoxin, aspirin and betablockers and other medications that may interfere with the study protocol, and high preoperative systolic or diastolic BP (over 160 mmHg and 90 mmHg, respectively).

Eligible patients were randomly divided into two groups: in the first group, intravenous TXA 15 mg/kg diluted in 100 ml normal saline was given, infused in 10 minutes after induction. In the second group, 0.2 mg oral clonidine was given, 1 to 1.5 hours before surgery; this group also received 100 ml intravenous normal saline, 10 minutes after induction. Randomization was performed using a table of random numbers. Both surgeon and patient were blind to the group allocation. The operations were performed by a single surgeon and by the same method (cold dissection without using a shaver). The same anesthesia protocol was also used in the two groups: volume expansion by 3.0 ml/kg of isotonic crystalloid before anesthesia induction, premedication by IV fentany, l-2 µg/kg and midazolam 0.02 mg/kg 3 to 5 minutes before intubation, induction by propofol 2.0 mg /kg and atracurium (0.5 g/kg), and maintenance by propofol 100 µg/kg/min, remifentanil 0.1µg/kg/min and atracuriam. At the end of surgery, muscle relaxant was reversed by neostigmine 0.04 mg/kg and atropine 0.02 mg/kg. A 30-degree reverse Trendelenburg position was used for all patients during surgery. Epinephrine, 1/2000-soaked pledges, was used before the operation and also during surgery, when considered necessary based on the surgeon's preference. The Lund-MacKay score for paranasal computed tomography (CT) scans, duration of surgery (defined as the time between delivering the patient to the operation room and completion of the surgery), pre- and post-surgical hemoglobin (Hb) level, heart rate (HR) and BP (every 15 minutes), quality of surgical field based on Boezzart's scale, and surgeon satisfaction level based on Likert scale were recorded in both groups. Likert scale was scored from 1 to 5 (very bad, bad, average, good and excellent) and Boezzart's scale was as follows: 0 for no bleeding, 1 for minor bleeding that does not need suctioning, 2 for minor bleeding that needs infrequent suctioning, 3 for minor bleeding that requires repeated suctioning when the surgical field is threatened by bleeding a few seconds after removing the suction, 4 for moderate bleeding that requires repeated suctioning when the surgical field is threatened by bleeding directly after removal of the suction, and 5 for severe bleeding that requires continuous suctioning when bleeding severely threatens the surgical field and the severity of the bleeding is greater than the extent that can be resolved by suctioning. The Hb level was once again measured 6 hours after the operation. The collected data were analyzed using SPSS (ver. 22), and a t-test and Chi-square test were used wherever appropriate. The significance level was set at P<0.05.

## Results

A total of 52 patients completed the study; 27 (51.9%) males and 25 (48.07%) females. Thirteen (25%) patients had chronic sinusitis without polyposis whereas 39 (75%) had chronic sinusitis with polyposis. Twenty-two (42.3%) cases were in the TXA group and 30 (57.7%) in the clonidine group. Regarding demographic characteristics, baseline hemodynamic data and preoperative Hb level, no significant difference was recorded between the two groups.

Based on the Chi-square test, the number of chronic polyposis and chronic sinusitis cases did not significantly differ between the two groups (P=0.746). The Lund-MacKay score showed no meaningful difference between the clonidine and TXA group based on the t-test (P=0.862). The mean pre- and postoperative Hb level also revealed no statistically meaningful difference between the two groups (P=0.930 and P=0.325, respectively). The difference in Hb level drop was again insignificant between the two groups (P=0.345). The comparison of mean arterial pressure (MAP) and HR between the two study groups demonstrated no statistically significant difference. The demographic and perioperative data of patients in both groups is presented in (Table.1).

**Table 1:** Comparison of demographic and perioperative data between the two studied groups

**Variable**	**Tranexamic acid group**		**Clonidine group**		**P-value**
	Mean	Standard Deviation	Mean	Standard Deviation	
Age (yr)	43.41	15.48	39.86	8.94	0.345
Lund-McKay score	14.23	7.22	13.85	7.78	0.862
Preoperative Hb (g/dl)	14.31	1.93	14.27	1.66	0.930
Postoperative Hb (g/dl)	13.21	1.90	12.67	1.59	0.325
Duration of surgery (min)	140.45	63.07	143.28	67.43	0.916
Hb drop (g/dl)	1.14	1.04	1.40	0.74	0.345
BMI	24.29	4.22	26.59	5.56	0.127

The quality of the surgical field based on Boezzart's scale showed no significant difference between the two groups at 30-, 60-, 90-, 120-, 150-, 180-, 210-, 240- and 270-minute time points. Surgeon satisfaction of the surgical field during surgery also showed no significant difference between the clonidine and TXA groups based on the five-point Likert scale (P=0.757, Table.2).

**Table 2:** Surgeon satisfaction based on Likert scale

	**Tranexamic acid group**		**Clonidine group**		**P-value**
	No.	%	No.	%	
Very bad	1	4.5	0	0.0	0.757
Bad	0	0.0	0	0.0	
Moderate	5	22.7	6	20.0	
Good	14	63.6	22	73.3	
Very good	2	9.1	2	6.7	

Based on Boezzarts's scale, surgical field quality was associated with systolic BP only at the 30-min time point (P=0.001). No association was found between either systolic or diastolic BP at the other time points. Moreover, the mean anesthesia time (P=0.859) and mean surgical time (P=0.880) were not significantly different between the clonidine and TXA groups. One patient in the clonidine group experienced cerebrospinal fluid (CSF) leakage, which was promptly managed. No signs or symptoms of thromboembolic events were observed in either group.

## Discussion

Blood-loss reduction and increased visibility of the surgical field are of great importance to surgeons to increase the success rate of endoscopic sinus surgery (14). So far, several methods have been used to decrease surgical field bleeding; e.g. reverse Trendelenburg position up to 30°, topical mucosal vasoconstrictors, and controlled hypotension (3). Using topical and systemic steroids preoperatively has also been suggested for this purpose, intended to decrease inflammation, shrink nasal polyps and reduce blood loss (6).

Additionally, the anesthesiologist also has a major role in improving surgical conditions to make visualization of the surgical field better. In this way, surgical complications such as optic nerve or orbit damage, CSF leakage, or even internal carotid artery injury can be further avoided (3), which could lead to reduced surgical costs and lower rates of anesthesia-related comorbidities (6).

Furthermore, MAP reduction during general anesthesia has been assumed to minimize intraoperative blood loss. However, it should be kept in mind that in addition to MAP, capillary blood flow and venous pressure may affect the extent of surgical blood loss, although MAP is the only variable which can be simply measured (15).

*Clonidine and recent study observations*

Clonidine is an alpha2 blocker that reduces BP both centrally and peripherally; it increases coagulation due to platelet activation (16), thus improving surgical field visualization by reducing bleeding (4). As a central effector, clonidine can reduce postoperative pain, nausea and vomiting. It also lowers the systemic BP by constriction of peripheral blood vessels, and so decreases the blood flow to the nasal mucosa.

Many recent studies on the effect of clonidine in FESS concluded that clonidine reduces arterial BP and increases surgeon visualization during surgery. In addition, they have reported shorter surgical time and reduced bleeding (6).

In a study by Cardesin et al. a better surgical field and lower morbidity of patients undergoing FESS was reported in the clonidine group (17). Turgul et al. also showed that premedication with oral clonidine provides a more vivid view of the surgical field, decreasing blood loss while significantly increasing surgeon satisfaction when FESS is performed due to nasal polyposis (6).

In a study by Mohammadi et al., clonidine premedication resulted in better control of bleeding in bimaxillary orthognathic surgery, shorter surgical time and enhanced surgical outcomes (4). Puthenveettil et al. compared oral premedication with 50 mg metoprolol and 300 µg of clonidine; the latter provided a better operative field during FESS (15). The same results were reported by Wawrzniak et al. when comparing premedication with clonidine and midazolam before FESS (8). In the study by Mohseni and Ebneshahidi, premedication with 0.2 mg oral clonidine administered 90 minutes before surgery, effectively reduced bleeding during FESS (7). In another similar study by Wawrzniak et al. better surgical field quality along with a more favorable hemodynamic profile was achieved for clonidine compared with melatonin (18).

*Tranexamic acid and recent study observations*

The positive role of TXA has been proven in the treatment of digestive and urinary system bleeding, primary amenorrhea, thrombo- cytopenia, hemophilia, and von Willebrand disease. In addition, it has been applied topically in spinal surgery, in the treatment of recurrent traumatic hyphema, total knee arthroplasty and cardiac surgery (2). TXA prevents fibrinolysis by blocking the lysine-binding site of plasminogen to fibrin. In cardiac surgery, using an intravenous (IV) bolus dose of 10 mg/kg TXA followed by an infusion of 1.0 mg/kg/hour during extracorporeal circulation can increase the therapeutic plasma concentration of TXA to 5–10 mg/ml. 

The therapeutic concentration of TXA maintains for approximately 3 hours (1).

Nevertheless, certain side effects have been reported for TXA, including nausea, vomiting and possibly arterial or venous thrombosis. Although theoretically possible, the risk of thrombosis has not yet been verified in any randomized controlled trials. 

Alimian and Mohseni reported nausea and vomiting in less than 15% of the TXA group, indicating no significant difference from placebo (1).

The study by Choi et al. showed that during bimaxillary osteotomy, a single intravenous dose of TXA 20 mg/kg administered preoperatively resulted in significant reduction of intraoperative bleeding (10). The same results were reported by Eftekharian et al. in orthognathic surgery (10). However, the use of 0.05% TXA in an irrigant fluid demonstrated no meaningful decrease in intraoperative bleeding compared with placebo during orthognathic surgery (11). In contrast, the administration of 15 mg/kg of IV TXA significantly reduced intraoperative bleeding volume compared with placebo in the study by Nuhi et al. No thrombotic events or alteration in coagulation parameters were encountered postoperatively, and no meaningful difference in the rate of nausea and vomiting was mentioned between the two groups (14).

Alemian et al. showed that IV TXA decreased the amount of bleeding effectively as well as improving the surgical field during FESS (1). Jabalameli et al. and Yaniv et al. reported similar results using topical and oral TXA in FESS (3). In contrast, no significant effect was achieved for the adjuvant administration of intravenous TXA in reducing bleeding and improving the surgical field during FESS in Langille et al. study (2). The effect of TXA in 200 patients undergoing endoscopic nasal surgery was evaluated by Chhapola et al; a 72% decrease in intraoperative hemorrhage was achieved in these patients (19).

Moise et al. also reported around a 50% decrease in total blood loss both intraoperatively and postoperatively by TXA (10 mg/kg) (20). In the study by El Shal and Hasanein, TXA and aminocaproic acid effectively reduced bleeding during FESS and improved the surgical field visualization, resulting in increased surgeon satisfaction, although no meaningful difference between the two drugs were found (3).

In a study by Abbasi et al., the administration of 15 mg/kg IV TXA was more effective than 5 mg/kg TXA in improving the surgical field visualization and hemostasis and surgeon satisfaction, decreasing the operative time and blood loss during FESS with no significant side effects (21).

Taken together, in the majority of studies performed to examine these matters, clonidine and TXA were more effective than placebo in reducing intraoperative bleeding and in providing a better surgical field during FESS. To the authors' knowledge, no study has yet been conducted comparing the effect of clonidine and TXA together; our study was designed to evaluate and compare the premedication effect of oral clonidine and IV TXA on blood loss and the surgical field quality during FESS to find out which drug better improves surgical field visualization. Our findings revealed no statistically significant difference between these two medications. Therefore, when one drug is contraindicated, the other drug can be safely applied.

The main limitation of the current study was the small sample size and the lack of a placebo (control) group. Future studies on a larger population and with a control group are advised to further confirm our findings. Additionally, the combination effect of these two drugs could be compared with the effect of each drug individually.

## Conclusion

Premedication with oral clonidine and intravenous TXA has the same effect on bleeding during FESS, surgical field visualization and surgeon satisfaction.
